# The optimal oxytocin infusion rate for preventing uterine atony during cesarean delivery in elderly parturients with prior history of cesarean delivery

**DOI:** 10.3389/fphar.2023.1211693

**Published:** 2023-07-28

**Authors:** Li Ying Wang, Jin Wang, Jin Hua Dong, Ze Peng Ping, Xin Zhong Chen, Chang Na Wei

**Affiliations:** ^1^ Department of Anesthesia, Jiaxing University Affiliated Women and Children Hospital, Jiaxing, China; ^2^ Department of Obstetrics, Jiaxing University Affiliated Women and Children Hospital, Jiaxing, China; ^3^ Department of Anesthesia, Women’s Hospital, Zhejiang University School of Medicine, Hangzhou, China

**Keywords:** oxytocin, maternal age, cesarean delivery, drug delivery, postpartum hemorrhage prevention

## Abstract

**Background:** An estimate of 90% effective dose (ED90) of oxytocin infusion has already been proved effective in non-laboring parturients. However, the requirements of oxytocin for elderly parturients with prior history of cesarean delivery (CD) may be higher. The aim of this study was to find the optimum oxytocin infusion rate for preventing uterine atony during CD in elderly parturients with prior history of CD.

**Method:** We performed a randomized, double-blinded study in 120 healthy elderly parturients with prior history of CD scheduled for elective CD under combined spinal–epidural (CSE) anesthesia. Participants were treated with oxytocin infusion randomly at the rates of 0, 4, 8, 12, 16, or 20 IU h^−1^ after the delivery of infants. Following oxytocin administration, a blinded obstetrician evaluated the uterine tone (UT), verbally describing it using numerical scales (0–10: 0, no UT; 10, optimal UT) as either adequate or inadequate at the time intervals of 3, 6, and 9 min. Maternal adverse effects, requirements for additional uterotonic agents, delivery–placenta delivery time (PD), and estimated blood loss (EBL) were recorded.

**Results:** The 50% effective dose (ED50) and 90% effective dose (ED90) of oxytocin infusion were 14.6 IU h^−1^ (95% confidence interval 12.0–18.4 IU h^−1^) and 27.7 IU h^−1^ (95% confidence interval 22.5–39.4 IU h^−1^), respectively. As the rate of infusion was increased in parturients, the rescue oxytocin dose and delivery-PD time were decreased. Parturients who received 0 IU h^−1^ oxytocin at 3, 6, and 9 min obtained lower UT scores than those who received 16 and 20 IU h^−1^ oxytocin (*p* < 0.05, respectively). No significant differences were observed among groups in EBL and maternal adverse effects.

**Conclusion:** The infusion rate of oxytocin at 14.57 and 27.74 IU h^−1^ produces adequate UT in 50% and 90% of elderly parturients with prior history of CD, respectively. An oxytocin infusion rate of 27.7 IU h^−1^ is suggested to be the optimal dose for preventing uterine atony during CD in elderly parturients with prior history of cesarean delivery.

**Clinical Trial Registration:** [https://www.chictr.org.cn/bin/project/edit?pid=62489], Identifier: [ChiCTR2000038891].

## 1 Introduction

The number of parturients who give birth at an advanced maternal age (≥35 years) has been increasing these days worldwide (Chan and Lao, 2008; Matthews and Hamilton, 2014; Office for National Statistics for Births in England and Wales, 2018). It has been verified that advanced maternal age increases the likelihood of intrapartum complications of cesarean section and postpartum hemorrhage (PPH) (Lao et al., 2014; Kanmaz et al., 2019). Therefore, elderly parturients with prior history of cesarean delivery (CD) comprise a critical proportion of CD.

When a caesarean section is performed, it is common practice in hospitals to give the parturient oxytocin after the delivery of the fetus. A rapid oxytocin I.V. bolus, even in small dose, is linked to either mild or severe adverse effects, such as, hypotension, myocardial ischemia, and hemodynamic collapse, especially among women with various cardiac conditions. Oxytocin infusion is advantageous in maintaining uterine tone (UT) throughout the operative and postoperative periods because of oxytocin’s short half-life. Thereby, numerous practitioners would rather infuse than inject to administer a certain dose of oxytocin.

For elective CD, the minimum effective dose of I.V. oxytocin administration has been determined by a few studies. The effective doses of ED90 oxytocin infusion for non-laboring parturients in elective CD were 16.2 and 17.4 IU h^−1^ in order for adequate UT (George et al., 2010; Lavoie et al., 2015). However, the dose of intravenous oxytocin infusion following delivery in CD remains inconclusive based on parturients’ situation, and consequently, it is inappropriate to apply a universal dose to all cases (Heesen et al., 2019). An *in vitro* study indicated that the occurrence rate of multiphasic spontaneous myometrial contractions had likely been increased with increasing parturients’ maternal age and their decreasing spontaneous activity (Smith et al., 2008). Bobrowski and Bottoms (1995) performed a retrospective review of over 8 years in 9,556 cases of delivery of singleton pregnant women, with ages ranging from 20 to 29 or above 35 years, and showed the highest age-related increases in oxytocin use (1.7 times). Furthermore, prior CD has been shown to predispose parturients to an increased risk of maternal morbidity, especially PPH (Ma et al., 2016). The aim of this dose–response research was to find the optimum oxytocin infusion rate for preventing uterine atony during CD in elderly parturients with prior history of CD.

## 2 Materials and methodologies

### 2.1 Ethical approval and consent to participate

The Ethical Committee of Jiaxing University Affiliated Women and Children Hospital (Jiaxing, China) approved this study on 21 July 2020 (No. 2020-46), and it was conducted in conformity with the Declaration of Helsinki. Each participant signed and handed in a written informed consent. The experiment was registered in the Chinese Clinical Trial Registry ahead of participant enrollment (registration no. ChiCTR2000038891; date of registration, 9 October 2020).

### 2.2 Participants

In this randomized, double-blind, dose-finding research, the inclusion criteria consisted of Physical Status II by the American Society of Anesthesiologists, age of 35–50 years, singleton pregnancy, gestational age of ≥37 weeks, elective CD with a planned Pfannenstiel incision, and planned combined spinal–epidural (CSE) anesthesia. All the parturients were treated with secondary CD. Prior history of CD was the primary indication of elective CD. Exclusion criteria consisted of known allergy to oxytocin, severe obstetric diseases (e.g., pre-eclampsia or pregnancy-induced hypertension), ruptured membranes, BMI ≥ 40 kg/m^2^, active labor, prior uterine fibroid resection, and risk factors of postpartum hemorrhage (e.g., macrosomia, placenta previa, history of uterine atony and postpartum bleeding, multiple gestations, uterine fibroids, polyhydramnios, or bleeding diathesis).

After enrollment, participants were randomized using the Microsoft Excel RAND function to determine study subject allocation. Group assignments were kept in opaque and sealed envelopes so as to guarantee the blinding of the research members. Participants were treated with oxytocin infusion randomly at the rates of 0, 4, 8, 12, 16, or 20 IU h^−1^, to be given from the delivery of the fetus to the end of the caesarean surgery. On the day of surgery, 50 ml syringes were filled with different units of oxytocin according to the randomization assignment by an anesthetist who was not a member of the study (e.g., when a participant was treated randomly at the rate of 4 IU h^−1^, 4 IU oxytocin and normal saline were drawn into the syringe of a total volume of 50 ml). All research participants were treated with different total oxytocin doses at the same infusion level (50 ml h^−1^). The patients, obstetricians, and anesthetists who were involved in the study were all blinded to the real oxytocin doses.

### 2.3 Anesthesia and data collection

Upon entry into the operation room, an 18-gauge intravenous (I.V.) cannula was inserted in the dorsum of one hand of the patient, and 30 min before the placement of CSE anesthesia, Ringer’s lactate infusion at 10 ml/kg/h was started. The heart rate, 5-lead electrocardiogram, pulse oximetry, and non-invasive blood pressure (NIBP) were monitored. In surgery, the patient’s heart rate and NIBP were measured at 3-min intervals. A CSE technique was performed at the L3/4 interspace via the needle-through-needle strategy. On confirming the aspiration of clear cerebrospinal fluid, 9–12 mg of 0.5% hyperbaric bupivacaine was applied to induce spinal anesthesia for a block height at the T5 dermatome according to parturients’ abdominal girths and vertebral column lengths by using the regression equation: Y_T5_ = 0.074X_1_ − 0.022X_2_ − 0.017 (where Y_T5_ = 0.5% hyperbaric bupivacaine volume for T5 block level, X_1_ = vertebral column length, and X_2_ = abdominal girth) (Wei et al., 2017). In the whole operation process, supplemental oxygen was administered to all parturients via a nasal cannula at the rate of 3 L/min. An experienced anesthetist who was not involved in the research was in charge of the intraoperative fluid management.

The cases where the systolic blood pressure was lower than 90 mmHg or where there was a decrease of more than 20% from the baseline were defined as hypotensive and were then treated with 6 mg ephedrine or 100 μg phenylephrine intravenously when hypotension occurred with or without bradycardia (HR < 50 beats min^−1^). The cases where the HR was 120 beats min^−1^ or above were defined as tachycardiac and were then treated with 1 mg/kg esmolol hydrochloride.

After the delivery of the infant, all parturients were given different total doses of oxytocin infusion at the rate of 50 ml h^−1^ that was previously prepared according to the randomization scheme. In the research, all the parturients, obstetricians, and anesthetists were blinded to the actual dose of oxytocin. After the initiation of oxytocin infusion, the attending obstetrician made an assessment of the UT through manual palpation of the parturient’s uterus at the time intervals of 3, 6, and 9 min. The obstetrician provided two subjective assessments of UT at each time point: 1) adequate or inadequate UT and 2) a UT score (UTS) using a verbal numerical scale score (0–10: 0, no UT; 10, optimal UT). The obstetrician rated the UT as “firm” and an UTS ≥7 (adequate) or as “boggy” and an UTS <7 (inadequate). An I.V. infusion of 3 IU of oxytocin was administered when the UT was judged as inadequate at 3 min. Another I.V. infusion of 3 IU of oxytocin was administrated again when the UT was again judged as inadequate at 6 min. When the UT was still inadequate at 9 min, secondary uterotonic agents (0.25 mg of intramuscular carboprost tromethamine, 0.2 mg of intramuscular methylergonovine maleate, or 800–1,000 mg of rectal misoprostol) were administered upon the obstetrician’s request. The oxytocin infusion was continued until the end of cesarean surgery.

### 2.4 Outcome

The primary outcomes were the results of the obstetrician’s judgment of the UT at 3 min as either adequate or inadequate following the first oxytocin administration. The secondary outcomes comprised the estimated blood loss (EBL) (estimated through measuring the suctioned blood and the blood weight on surgical swabs), UTS, hemoglobin (Hb) and hematocrit (HCT) levels (within 30 min after the operation and on postoperative day 2), rescue doses of oxytocin or alternative uterotonic agents, delivery–placenta delivery time (PD), and side effects in connection with oxytocin (e.g., nausea, tachycardia, vomiting, hypotension, flushing, and chest pain). The demographic characteristics, antepartum hemoglobin level, fetal weight, suctioned amniotic fluid volume, and surgical duration were also recorded.

### 2.5 Statistical analysis

To evaluate whether the data complied with the normal distribution of variance, the Kolmogorov–Smirnov test together with normality plots was adopted. Then, for the assessment of the normally distributed data, one-way analysis was applied; as for pairwise comparisons, the *post hoc* Bonferroni test was conducted. Furthermore, the Kruskal–Wallis test was carried out to test the non-normally distributed data, which were described as median (range). The Chi-square test was performed for the assessment of categorical variables, and the Fisher exact test was instead performed where it was necessary. The correlation of the total dose of oxytocin infusion and UTS was determined using linear regression analysis. An effective oxytocin infusion rate (success) was defined as the rate at which adequate UT was provided from the initiation of oxytocin infusion to the end of CD, in the absence of administration of additional uterotonic agents. The data for successful responses for each infusion rate were used to plot a sigmoid dose–response curve. In the end, the SPSS version 19.0 (SPSS Inc., Chicago, IL, United States) was adopted for statistical analyses, where *p* < 0.05 was regarded as statistically significant.

Through the PASS (version 11.0.7; NCSS, LLC, Kaysville, Utah, United States), the Cochran–Armitage test was conducted to calculate the sample size on the basis of the preliminary data of the six groups at the oxytocin infusion rates of 0, 4, 8, 12, 16, and 20 IU h^−1^. The preliminary data showed that parturients with adequate UT took up the proportion of 0.05, 0.2, 0.3, 0.4, 0.55, and 0.7, respectively. Through the Z test with continuity correction and a significance level of 0.05, it was finally calculated that in total, a sample size of 48 patients, that is, eight patients in each group, would play 90% of the part in detecting a linear trend in the ratio of parturients who had adequate UT in the six groups. In view of potential dropouts, we raised the sample size to 120 parturients in total.

## 3 Results

**FIGURE 1:**
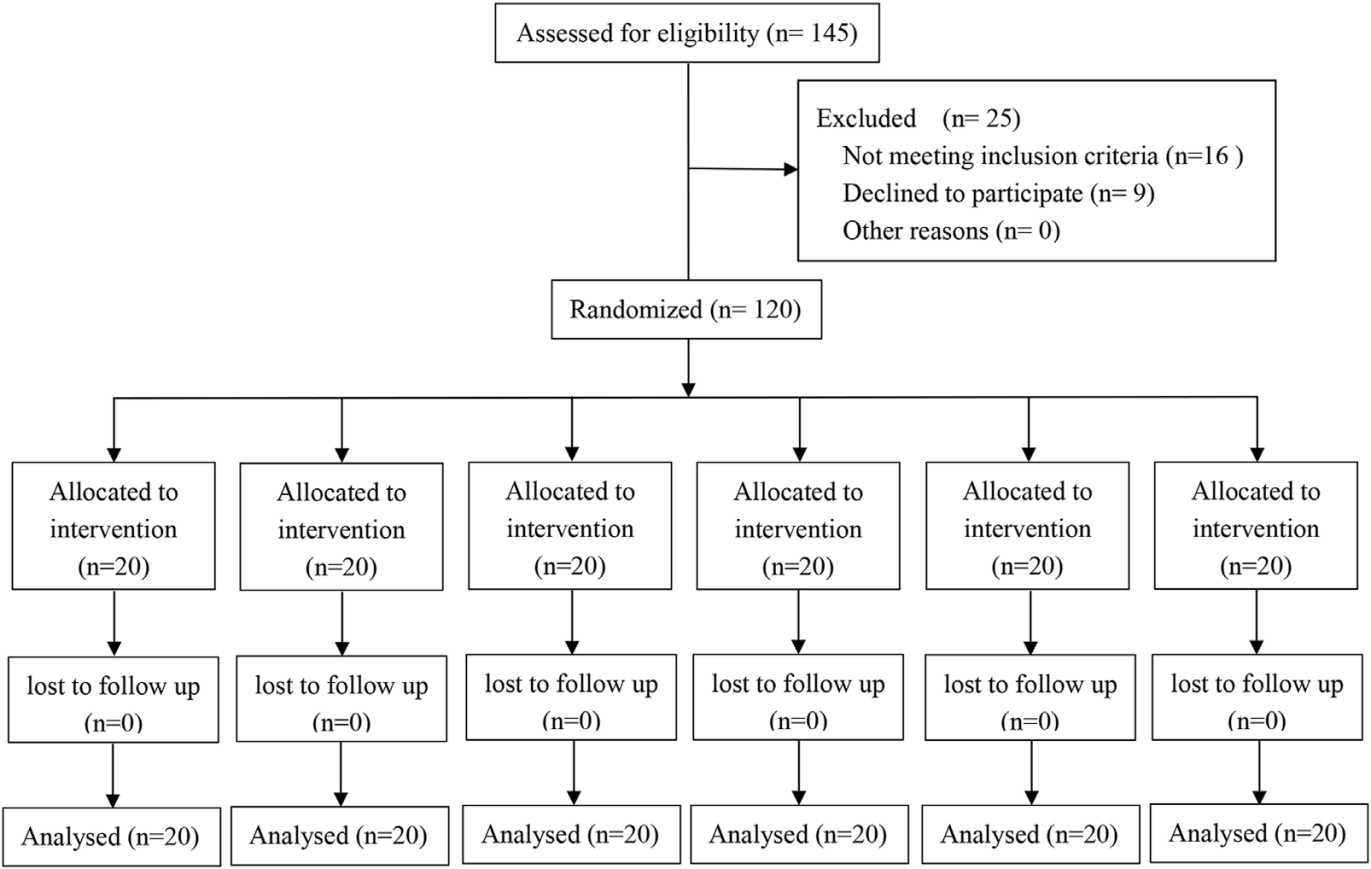
Study flow diagram.

Following the enrollment and randomization, a total of 120 parturients completed the study (Figure 1). The six groups did not show a significant baseline difference in the aspects of their demographic characteristics, preoperative Hb, preoperative HCT, or surgery time (Table 1).

**TABLE 1:** Demographic characteristics and obstetric data.

	0 IU h^−1^	4 IU h^−1^	8 IU h^−1^	12 IU h^−1^	16 IU h^−1^	20 IU h^−1^	*p*-value
Age (yr)	37.2 ± 1.6	37 ± 1.1	38.2 ± 2.8	38.5 ± 2.1	37.4 ± 2.3	37.6 ± 2.1	0.166
Weight (kg)	71.9 ± 9.1	69.3 ± 8.0	71.1 ± 8.1	70.9 ± 5.8	70.0 ± 9.8	69.0 ± 9.1	0.880
Height (cm)	159.4 ± 3.1	159.8 ± 4.7	160.6 ± 3.3	160.4 ± 2.5	160.5 ± 4.3	158.8 ± 4.1	0.583
BMI (kg m^−2)^	28.27 ± 3.1	27.1 ± 2.7	27.5 ± 2.5	27.5 ± 2.1	27.2 ± 3.9	27.3 ± 3.9	0.847
Parity	2 (2–3)	2 (2–3)	2 (2–3)	2 (2–3)	2 (2–3)	2 (2–3)	0.992
Gravidity	3.6 ± 0.9	3.3 ± 1.0	4.2 ± 1.0	3.6 ± 1.4	3.7 ± 1.3	3.3 ± 1.2	0.132
Gestational age (week)	39 (38, 39)	38.5 (38, 39)	39 (38, 39)	39 (38, 40)	39 (38, 39)	39 (38, 39)	0.623
Previous CS history	1 (1, 1)	1 (1, 1)	1 (1, 1)	1 (1, 1)	1 (1, 1)	1 (1, 1)	1.0
Amniotic fluid volume (ml)	625.0 ± 326.7	632.5 ± 199.5	607.5 ± 208.6	642.5 ± 355.9	702.5 ± 307.6	679.0 ± 245.0	0.897
Preoperative Hb (g L^−1^)	110.2 ± 12.32	117.2 ± 12.2	112.4 ± 14.5	118.5 ± 10.2	113.3 ± 13.6	109.2 ± 10.7	0.116
Preoperative HCT (%)	33.4 ± 3.3	34.6 ± 3.3	34.0 ± 4.0	35.3 ± 2.5	34.0 ± 3.5	33.1 ± 2.6	0.307
Fetal weight (g)	3296.8 ± 415.6	3461.5 ± 319.3	3369.0 ± 313.1	3382.3 ± 416.2	3355.3 ± 353.6	3372.5 ± 393.6	0.842
Surgery time (min)	51.1 ± 16.7	46.1 ± 11.9	51.1 ± 11.4	46.1 ± 10.1	46.7 ± 10.5	48.5 ± 10.9	0.580

Data are presented as mean ± standard deviation or median (range). CS, cesarean section; Hb, hemoglobin; HCT, hematocrit.

**FIGURE 2:**
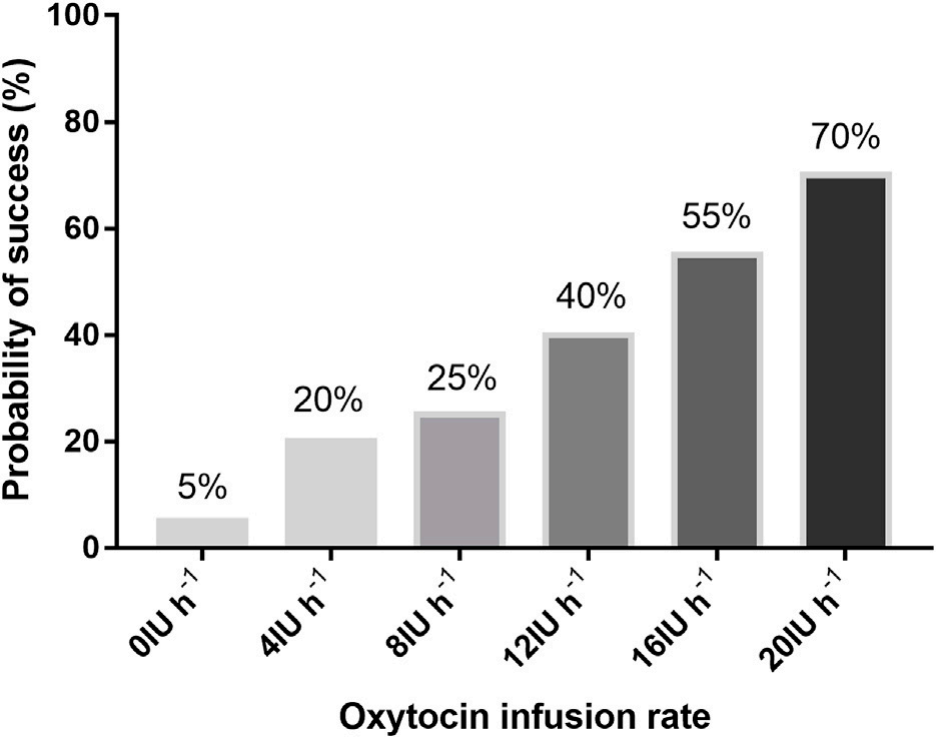
Proportion of patients with adequate uterine tone with different infusion rates of oxytocin.

**FIGURE 3:**
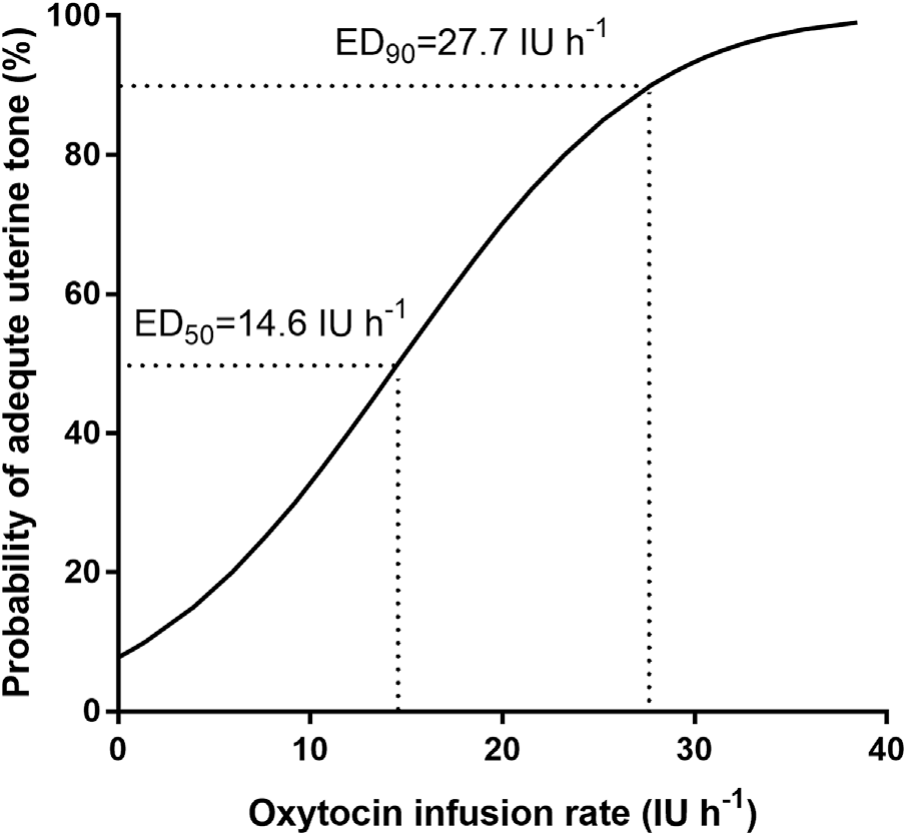
Dose–response curve of oxytocin infusion for preventing uterine atony: regression plot of the probit value vs. the infusion rate of oxytocin. The 0.5 and 0.90 y-intercepts indicate the ED50 and ED90, respectively.

The percentage of parturients with adequate UT at 3 min after oxytocin infusion is shown in Figure 2. The dose–response curve of oxytocin to prevent uterine atony is shown in Figure 3. The estimated ED50 and ED90 were 14.6 (95% CI 12.0–18.4) IU h^−1^ and 27.7 (95% CI 22.5–39.4) IU h^−1^, respectively.

**FIGURE 4:**
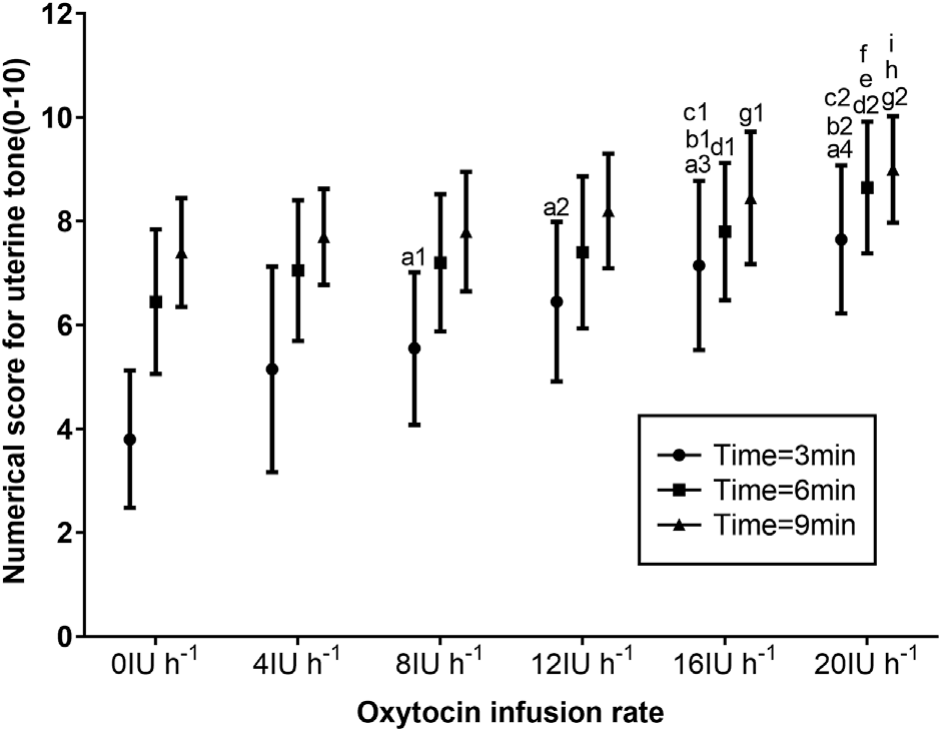
Numerical scores for UT according to the manual assessment by the obstetrician using a numerical scale of 0 (absent UT) to 10 (optimal UT) for groups receiving 0, 4, 8, 12, 16, and 20 IU h^−1^ oxytocin. Data are presented as mean ± standard deviation. ^a1^ *p* = 0.01, ^a2^ *p* = 0.001, ^a3^ *p* = 0.001, and ^a4^ *p* = 0.001 vs. 0 IU h^−1^ oxytocin group; ^b1^ *p* = 0.002 and ^b2^ *p* = 0.001 vs. 4 IU h^−1^ oxytocin group; and ^c1^ *p* = 0.026 and ^c2^ *p* = 0.001 vs. 8 IU h^−1^ oxytocin group at 3 min after oxytocin infusion. ^d1^ *p* = 0.031 and ^d2^ *p* = 0.001 vs. 0 IU h^−1^ oxytocin group; ^e^ *p* = 0.004 vs. 4 IU h^−1^ oxytocin group; and ^f^ *p* = 0.015 vs. 8 IU h^−1^ oxytocin group at 6 min after oxytocin infusion. ^g1^ *p* = 0.045 and ^g2^ *p* = 0.001 vs. 0 IU h^−1^ oxytocin group; ^h^ *p* = 0.004 vs. 4 IU h^−1^ oxytocin group; and ^i^ *p* = 0.001 vs. 8 IU h^−1^ oxytocin group at 9 min after oxytocin infusion.

The UTSs after oxytocin infusion at 3, 6, and 9 min are presented in Figure 4. When compared with the UTSs of those who received 8, 12, 16, and 20 IU h^−1^ oxytocin (*p* < 0.05), the UTSs of parturients receiving 0 IU h^−1^ were significantly lower at 3 min. Parturients receiving 16 and 20 IU h^−1^ had significantly higher UTSs at 3 min than those receiving 0, 4, and 8 IU h^−1^ oxytocin (*p* < 0.05). When compared with the UTSs of those receiving 0 IU h^−1^ (*p* < 0.05), the UTSs of parturients receiving 16 IU h^−1^ were significantly higher at 6 and 9 min. When compared with the UTSs of those receiving 0, 4, and 8 IU h^−1^ oxytocin (*p* < 0.05), the UTSs of parturients receiving 20 IU h^−1^ were significantly higher at 6 and 9 min (Figure 4).

The secondary outcomes are summarized in Table 2. The rescue oxytocin dose and delivery-PD time decreased with increasing oxytocin infusion dose (*p* < 0.05) (Table 2). No statistically significant differences were observed in terms of total I.V. crystalloid volume, EBL, and supplemental second-line uterotonic agents between the different groups. PPH was not observed in the study, and none of the parturients required a blood transfusion perioperatively.

**TABLE 2:** Secondary outcomes.

	0 IU h^−1^	4 IU h^−1^	8 IU h^−1^	12 IU h^−1^	16 IU h^−1^	20 IU h^−1^	*p*-value
Estimated blood loss (ml)	401.5 ± 121.6	326.5 ± 133.3	318.0 ± 96.3	315.0 ± 84.3	324.0 ± 101.6	323.5 ± 136.4	0.273
Hb within 30 min after the operation (g L^−1^)	105.4 ± 14.4	113.0 ± 11.7	106.8 ± 12.5	114.0 ± 13.8	107.0 ± 13.1	105.2 ± 13.9	0.134
HCT within 30 min after the operation (%)	32.1 ± 4.1	33.8 ± 3.3	32.5 ± 3.3	34.2 ± 3.8	32.4 ± 3.5	32.5 ± 4.0	0.357
Hb on postoperative 1 day (g L^−1^)	102.6 ± 14.0	111.0 ± 11.5	105.9 ± 11.6	112.7 ± 13.4	106.1 ± 12.7	104.9 ± 13.0	0.116
HCT on postoperative 1 day (%)	31.7 ± 3.9	33.8 ± 3.6	32.4 ± 3.3	34.0 ± 3.8	32.4 ± 4.0	32.3 ± 3.8	0.310
Total I.V. crystalloid (ml)	1037.5 ± 205.1	992.0 ± 140.3	1007.5 ± 142.6	951.0 ± 134.2	992.5 ± 192.8	1000.0 ± 259.6	0.805
Delivery-PD time (s)	187.9 ± 51.5	160.6 ± 38.8	158.7 ± 47.1	150.8 ± 32.1	138.0 ± 42.1^a^	123.7 ± 43.6^a^	<0.001
Rescue oxytocin dose (IU)	5.0 ± 1.5	3.8 ± 2.4	3.6 ± 2.5	3.0 ± 2.8	2.4 ± 2.9^a^	0.9 ± 2.0^abc^	<0.001
Supplemental second line uterotonic agents [n (%)]	6 (30)	6 (30)	5 (25)	5 (25)	4 (20)	2 (10)	0.622

Data are presented as mean ± standard deviation or *n* (%). ^a^ *p* <0.05 vs. 0 IU h^−1^ oxytocin infusion group. ^b^ *p* < 0.05 vs. 4 IU h^−1^ oxytocin infusion group. ^c^ *p* < 0.05 vs. 8 IU h^−1^ oxytocin infusion group. PD, placental delivery; Hb, hemoglobin; HCT, hematocrit.

No statistical difference was observed in terms of hypotension incidence, tachycardia, vomiting, headache, nausea, chest distress, and flushing between the different groups (Table 3). The side effects reported were not aggravated and quickly alleviated after symptomatic treatment or after the end of oxytocin infusion.

**TABLE 3:** Oxytocin-related adverse events.

	0 IU h^−1^	4 IU h^−1^	8 IU h^−1^	12 IU h^−1^	16 IU h^−1^	20 IU h^−1^	*p*-value
Hypotension [*n* (%)]	3 (15)	1 (5)	1 (5)	2 (10)	3 (15)	3 (15)	0.776
Tachycardia [*n* (%)]	2 (10)	0 (0)	0 (0)	2 (10)	2 (10)	2 (10)	0.448
Headache [*n* (%)]	0 (0)	0 (0)	1 (5)	2 (10)	2 (10)	4 (20)	0.076
Nausea [*n* (%)]	3 (15)	0 (0)	2 (10)	1 (5)	3 (15)	2 (10)	0.330
Vomiting [*n* (%)]	3 (15)	0 (0)	1 (5)	1 (5)	0 (0)	1 (5)	0.227
Chest distress [*n* (%)]	2 (10)	2 (10)	1 (5)	1 (5)	2 (10)	3 (15)	0.885
Flushing [*n* (%)]	0 (0)	1 (5)	1 (5)	1 (5)	1 (5)	1 (5)	0.867

Data are expressed as *n* (%).

## 4 Discussion

This dose–response research obtained the primary finding that ED50 and ED90 of oxytocin infusion during caesarean delivery were 14.6 (95% CI 12.0–18.4) IU h^−1^ and 27.7 (95% CI 22.5–39.4) IU h^−1^, respectively, in elderly parturients with a prior history of CD. In addition, the rescue oxytocin dose and delivery-PD time decreased with the increasing oxytocin infusion dose. Parturients in the group of 20 IU h^−1^ oxytocin infusion were in need of the least rescue oxytocin bolus and delivery-PD time among the six groups. The ED90 of oxytocin infusion was deduced from the probit analysis and outside the dose range of this study; however, several previous studies have suggested that 30 IU h^−1^ of oxytocin infusion is safe during CD (Munn et al., 2001; Committee on Practice Bulletins-Obstetrics, 2017; Ghulmiyyah et al., 2017; Ahmadi, 2018). Therefore, we recommend 27.7 IU h^−1^ of oxytocin as the optimal dose to avoid uterine atony when elderly parturients who have had prior history of CD are going through a CD.

In the last decade, considerable research have been conducted as to how much and in what ways oxytocin should be administered at the third stage of CD (Heesen et al., 2019; Baliuliene et al., 2021; Phung et al., 2021). Although consensus regarding the optimal oxytocin dose and mode is lacking, adverse hemodynamic effects of 5 IU oxytocin bolus have been verified (Baliuliene et al., 2021). Thomas et al. (2007) found that 5 IU oxytocin bolus when compared with infusion resulted in significantly increased heart rates and decreased mean arterial pressure, but blood loss did not significantly vary. Miyoshi et al. (2021) came to the same conclusion and advised us to discard the I.V. bolus injection of oxytocin when cesarean section (CS) was performed, particularly when non-laboring women were going through an elective CS. Therefore, most practitioners would rather choose the way of infusion to administer oxytocin than use a bolus for safety considerations.

Previous studies have estimated that the ED90 of oxytocin as an infusion was 16.2 and 17.4 IU h^−1^ for preventing uterine atony in parturients scheduled for CD without labor (George et al., 2010; Lavoie et al., 2015). In this study, we found that the ED90 of an oxytocin infusion was raised to 27.7 IU h^−1^ in elderly parturients with prior history of CD. Patel et al. (2017) demonstrated that in comparison with 3-month-old mice, 8-month-old mice had less connexin-43 mRNA expression and oxytocin receptor in the myometrium, which was in collaboration with a more frequent yet shorter duration of spontaneous myometrial contractions and a weaker contractile reaction to oxytocin. Another animal study reached the conclusion that the myometrium of young laboring rats appear ready to contract to the maximum extent, while blunted contractions occur in the myometrium of older non-laboring rats and the contractile potential has to be stimulated (Elmes et al., 2015). Clinical research has also shown that elderly parturients required more oxytocin for preventing uterine atony (Bobrowski and Bottoms, 1995; Wei et al., 2020). Our previous study estimated the ED50 of oxytocin was greater in the parturients with a history of prior caesarean delivery than in the parturients without a history of prior caesarean delivery (Wei et al., 2021). Therefore, uterine scarring and advanced maternal age may be responsible for the increased requirements of oxytocin in elderly parturients with a prior history of CD.

In this study, we found that the UTSs at 3, 6, and 9 min were increased when the oxytocin infusion dose was increased. In the meanwhile, the UTS increased with increasing oxytocin infusion time in each group. Both the increase in the dose of oxytocin infusion and the extension of the time of oxytocin infusion will increase the concentration of oxytocin in the blood, which will enhance the maternal uterine contractions. Previous investigations have indicated that larger infusion doses of oxytocin turn out more productive than smaller doses to avoid potential remedies for PPH, especially in the case of CD (Roach et al., 2013). The results of the current study are consistent with previous findings.

Oxytocin is linked to hemodynamic side effects ranging from myocardial ischemia, arrhythmias, vomiting, hypotension, chest pain, nausea, headache, to flushing. However, such side effects have connections with oxytocin doses and its administration rates (Jonsson et al., 2010). There was no statistical difference observed in the oxytocin-related adverse events among the different dose infusion groups. This may be attributed to the small dose difference of only 4 IU h^−1^ between the groups in this study. The oxytocin-related side effects were not aggravated and quickly alleviated after symptomatic treatment or after the end of oxytocin infusion.

Limitations still exist with this research even though its findings have significant clinical implications. Above all, the analyses of the side effects at the time interval ≥3 min after oxytocin was administered may have been disturbed in participants who were treated with rescue oxytocin doses; despite this, inadequate UT and bleeding were not allowed to persist without remedies because of ethics. In addition, the ED90 of the oxytocin infusion dose was deduced from the probit analysis and outside the dose range of this study. However, we can conclude from previous studies that the administration of 27.7 IU h^−1^ oxytocin during CD was a safe clinical practice (Munn et al., 2001; Committee on Practice Bulletins-Obstetrics, 2017; Ghulmiyyah et al., 2017; Ahmadi, 2018). Although it was the senior attending obstetricians who conducted the evaluation of adequate UT in this research, there might still exist problems of variability and subjectivity with uterine palpation. Nevertheless, there are no reliable and objective methods of clinical UT assessment. Subjective assessments have been adopted in previous studies assessing UT during CD too (Qian et al., 2019; Drew et al., 2020).

In summary, we demonstrate that the ED50 and ED90 of oxytocin infusion in preventing uterine atony during elective CD were 14.6 and 27.7 IU h^−1^, respectively, in elderly parturients with prior history of CD. An oxytocin infusion rate of 27.7 IU h^−1^ is suggested to prevent uterine atony during CD in elderly parturients with prior history of CD. We suggest that guidelines for oxytocin administration should differentiate between elderly parturients with prior history of CD and non-laboring patients.

## Data Availability

All data generated or analysed during this study are included in this published article. Data analysed during this study may be available from the corresponding author upon reasonable request.
